# From situational interest and state curiosity to personal interest: developmental pathways and underlying mechanisms

**DOI:** 10.3389/fpsyg.2025.1700208

**Published:** 2025-12-15

**Authors:** Yuling Yan, Yilei Jia, Yuxin Li, Feiyi Liu, Mingxia Zhang

**Affiliations:** 1 Institute of Psychology, Chinese Academy of Sciences, Beijing, China; 2 Department of Psychology, University of Chinese Academy of Sciences, Beijing, China

**Keywords:** interest, curiosity, knowledge acquisition, self-relevance, rewards processing

## Abstract

Personal interest, an enduring inclination toward specific topics or domains, supports sustained engagement, deep learning, and positive affect. However, how transient motivational states such as situational interest and state curiosity develop into stable personal interest remains unclear. This review begins with a conceptual analysis of interest and curiosity, focusing on the similarities and differences between situational interest and state curiosity. Building on this foundation, it integrates perspectives from educational psychology, cognitive neuroscience, and motivational science to organize two developmental pathways to personal interest. The first begins with situational interest and progresses through triggered and maintained situational interest to emerging and well-developed personal interest. The second begins with state curiosity and advances through curiosity triggering, evaluation of information value and perceived competence, information seeking and iterative satisfaction, and finally well-developed personal interest. Across both pathways, rewards processing functions as a core mechanism, driven by the intrinsic rewards of knowledge acquisition and the motivational effects of autonomy and self-relevance. This framework offers an integrated account of how fleeting motivational states can be transformed into enduring personal interest.

## 1 Introduction

Personal interest refers to an individual’s enduring inclination to engage with a particular topic or domain, associated with the pursuit of knowledge, perceived value, and positive emotional experiences (Hidi and Renninger, 2020). It influences cognitive functioning and performance, serving as a driver of sustained and effective learning. Even in the absence of external incentives such as monetary rewards, food, or prizes, personal interest can motivate individuals to participate in activities (Deci and Ryan, 2008). Students who are interested in a subject or task tend to allocate more attention, show greater persistence, and acquire higher-quality knowledge compared to their less interested peers (Hidi and Renninger, 2020). In addition to its cognitive benefits, personal interest contributes to positive affect, including increased happiness and life satisfaction (Ryan and Deci, 2022). For adolescents, the development of personal interests has been associated with pleasure and fulfillment, improvements in physical and mental health outcomes (Krnjaić, 2020), and lower levels of negative affect such as stress, boredom, loneliness, and depressive symptoms (Li et al., 2019).

However, in everyday life, the process by which personal interest is formed is not always clear. Why do curiosity and initial interest in a topic sometimes lead to sustained exploration and long-term engagement, while at other times they fade quickly, leaving no motivation for further pursuit? These temporary affective–cognitive states, experienced in response to specific contextual, task-related, or object-related features, are referred to as situational interest (Hidi and Renninger, 2006) and state curiosity (Loewenstein, 1994). A central question is how these experiences can give rise to stable personal interest. This paper examines that transformation by first clarifying the concepts of interest and curiosity and analyzing similarities and differences between situational interest and state curiosity. It then proposes two developmental pathways through which these transient states may evolve into personal interest, analyzes their stages, contributing factors, and underlying mechanisms, and concludes with implications for future research.

## 2 Interest and curiosity

Interest and curiosity are related yet distinct motivational constructs that support attention, learning, and exploration. Interest is a psychological state involving focused attention and meaningful engagement with specific content (Hidi and Renninger, 2019). It is categorized into situational interest, a temporary state triggered by external stimuli (e.g., novelty, social interaction, choice), and personal interest, an enduring disposition characterized by prior knowledge, personal value, and positive affect (Renninger et al., 2019). Personal interest can develop from repeated experiences of situational interest. Furthermore, the relationship between them is not strictly one-directional but rather cyclical, a core feature of the four-phase model of interest development (Hidi and Renninger, 2006). This cyclical nature is possible because personal interest exists as an enduring disposition–a potential to reengage–rather than a constantly activated state. Even an individual with a well-developed personal interest requires a specific situational trigger, such as novel information, to activate that interest in the moment (Hidi and Renninger, 2020). This triggered experience is, in essence, a manifestation of situational interest, which explains how the “state” can be reactivated even when the “disposition” is already established. Curiosity is a motivational state that drives the acquisition of new information, particularly when an individual perceives a gap in their knowledge (Litman, 2005). This paper focuses on state curiosity, a transient desire to resolve uncertainty, as opposed to trait curiosity, which reflects a stable tendency toward exploration (Litman et al., 2010). To maintain a clear focus, it is important to state at the outset that the central aim of this review is to understand the developmental pathways originating from transient motivational states. Therefore, while both interest and curiosity have trait-like dispositions, our analysis is exclusively concerned with situational interest and state curiosity as the starting points for personal interest. Unless otherwise specified, any subsequent mention of “curiosity” in this manuscript refers to its state form.

Situational interest and state curiosity share several features: both are transient motivational states that promote focused attention and exploration (Hidi and Renninger, 2020), and both can activate the brain’s rewards system without external incentives (Murayama, 2022). However, four key differences distinguish the two constructs. First, their triggering factors differ. While both can be seen as responses to informational stimuli, we adopt a functional distinction for our framework. State curiosity is triggered by the internal recognition of a specific information gap that produces uncertainty (Loewenstein, 1994). This aligns with the “knowledge-deprivation hypothesis” which, although sometimes discussed in the interest literature (Rotgans and Schmidt, 2014), is conceptually centered on resolving a perceived lack of knowledge. In contrast, situational interest is primarily triggered by external stimulus characteristics, such as novelty, surprise, or the inherent coherence of the content that engages the individual (Silvia, 2008). Second, their information-seeking characteristics differ: curiosity is goal-directed and diminishes once the specific information gap is resolved, whereas interest motivates broader and more sustained engagement beyond immediate acquisition (Shin and Kim, 2019). Third, their emotional experiences differ. Curiosity may involve an initial aversive state of tension associated with “not knowing,” which is then relieved and experienced as rewarding upon resolution (Jepma et al., 2012). In contrast, while the initial trigger for situational interest might evoke complex emotions like surprise or even confusion, the subsequent process of engagement is predominantly characterized by positive affect (Donnellan et al., 2022; Hidi and Renninger, 2006). Finally, they involve different neural mechanisms: curiosity is associated with dopaminergic circuits that support rewards-seeking, whereas situational interest is linked to opioid systems that generate pleasure (Shin and Kim, 2019).

## 3 Pathways in personal interest formation: situational interest and state curiosity

**FIGURE 1:**
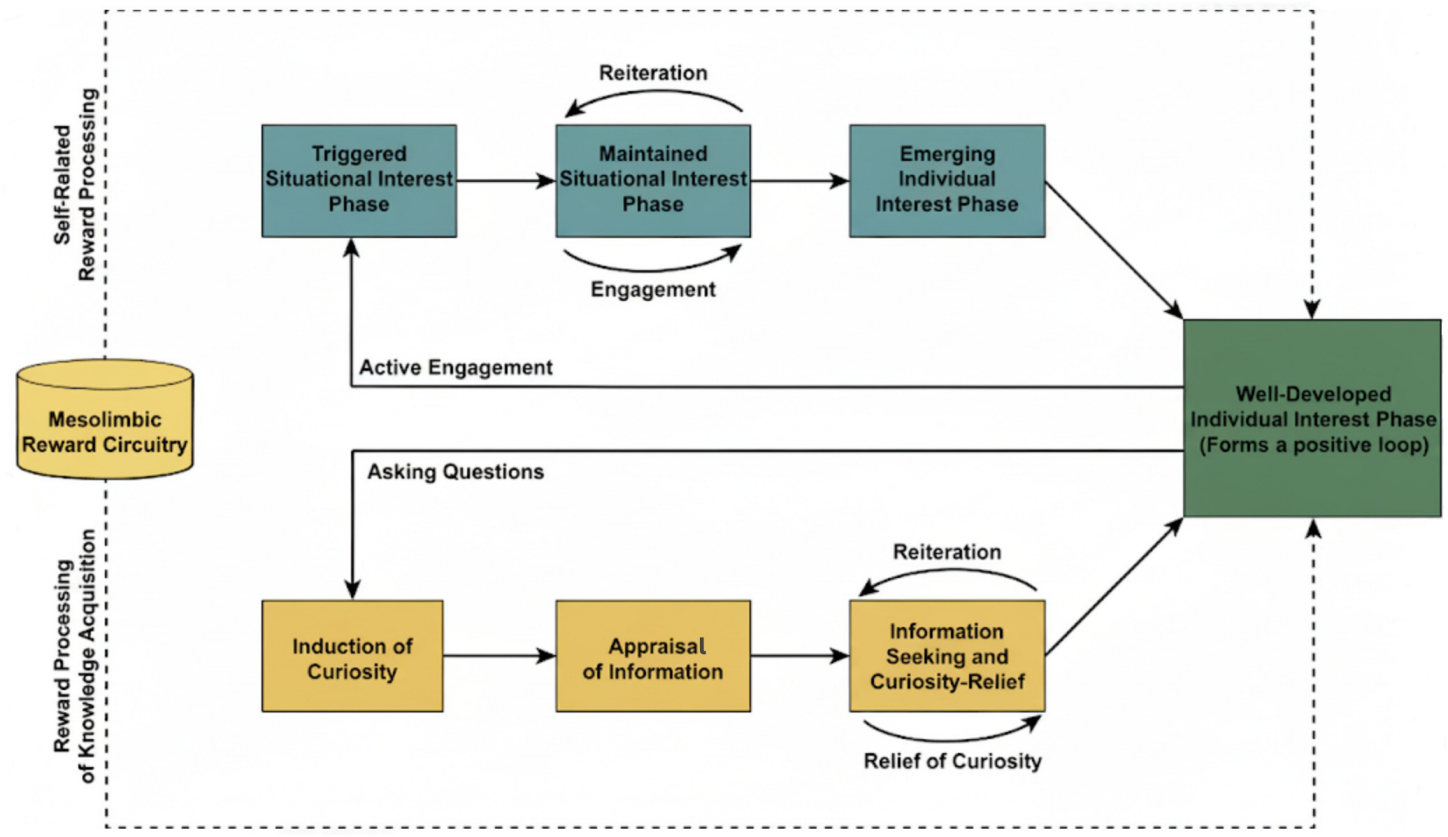
Framework of the pathways from situational interest and state curiosity to personal interest. The solid blue boxed path within the framework illustrates the stages from situational interest to personal interest, while the solid green boxed path illustrates the stages from state curiosity to personal interest. The outer dashed box represents the underlying mechanism of personal interest formation: the brain’s rewards circuitry.

While enduring interest can originate from transient experiences of situational interest and state curiosity, existing theoretical frameworks do not provide a comprehensive account of how these momentary states evolve into stable personal interest. Scholars from different disciplines have examined specific aspects of this developmental process: educational researchers have focused on the trajectory of interest development (Hidi and Renninger, 2020), whereas cognitive neuroscientists and computational modelers have emphasized the mechanisms underlying curiosity (Murayama, 2022). Situational interest and state curiosity are sometimes treated as interchangeable constructs, creating conceptual ambiguity. To address this, the present review integrates insights from education, psychology, and cognitive neuroscience to organize two developmental pathways, one from situational interest and the other from state curiosity, that may lead to the formation of personal interest. Figure 1 provides a visual representation of this integrated framework, illustrating the two proposed pathways and their shared underlying mechanism.

### 3.1 From situational interest to personal interest: interest enhancement through sustained engagement

This developmental pathway is largely based on the foundational four-phase model of interest development proposed by Hidi and Renninger (2006, 2020). The development from situational interest to personal interest reflects a progression from externally triggered engagement to internally driven motivation. In the early stage, interest may be elicited by external factors–such as instructional materials, environmental features, or social interactions. As engagement continues, the individual increasingly seeks related information autonomously, signaling a shift from passive exposure to active exploration. Over time, interest becomes internalized, emerging as a self-sustaining motive that supports long-term, self-directed engagement. Through this process, individuals acquire domain-specific knowledge and come to perceive its personal value, leading to more persistent involvement. This transformation can be delineated into four phases.

Phase 1: Triggered Situational Interest. An individual’s attention is captured by a source that is novel, surprising, or incongruous (Hidi and Renninger, 2006). These triggers may not necessarily produce immediate positive effect; emotions such as surprise, confusion, or even shock can serve to spark initial interest. The key function of this phase is to seize attention and motivate a preliminary engagement with the content. Such factors may be directly perceived or highlighted by others, and they often stimulate imagination and promote short-term information seeking (Palmer et al., 2017). For example, in educational contexts, teachers can foster situational interest by incorporating novel or surprising elements into classroom activities (Pressick-Kilborn, 2015). These factors can promote students’ attention to task utility (Harackiewicz et al., 2016) and to specific components of inquiry-based learning (Renninger et al., 2019). At this stage, a basic level of prior knowledge is sufficient to engage with and process the presented information. The role of others, such as educators or more knowledgeable peers, is often critical in this triggering process. Research has shown that interest can be effectively triggered when teachers intentionally structure activities with surprising or novel elements, or when they explicitly highlight the utility and relevance of a task (Harackiewicz et al., 2016). These forms of external support are crucial for directing attention and sparking the initial engagement that defines this phase (Renninger et al., 2019).

Phase 2: Maintained Situational Interest. This is a critical part of the four-phase model (Hidi and Renninger, 2006), as initial interest may fade or become sustained. To maintain engagement, it is important to provide repeated opportunities to interact with the content. Such ongoing interaction increases the likelihood that situational interest will persist and develop into a more enduring form. To maintain engagement, it is important to provide repeated opportunities to interact with the content. The transition from a fleeting triggered state to a more durable maintained one hinges on the individual beginning to appreciate the content’s personal relevance and value. It is during this phase that connections are formed between the topic and the individual’s own goals, experiences, or identity, providing the motivational foundation for sustained engagement. Affective experiences at this stage are predominantly positive, and learners benefit from sustained engagement. Repeated participation is important for individuals whose interest has been triggered but remains fragile and underdeveloped (Renninger et al., 2019). This phase underscores the role of instructional and contextual strategies in promoting the transformation of momentary situational interest into sustained engagement. Unlike the attention-capturing support in Phase 1, scaffolding in this phase aims to maintain engagement by helping learners build a sense of personal value and competence. Providing repeated opportunities for meaningful interaction and ensuring tasks are challenging yet manageable are key strategies for achieving this, thereby solidifying the transition toward a more enduring interest (Hidi and Renninger, 2006; Renninger et al., 2019).

Phase 3: Emerging Personal Interest. At this stage, individuals begin to independently pursue additional information on topics of interest, signaling the transition toward emerging personal interest. Their engagement becomes more purposeful; for instance, they may actively seek out specific resources like books or expert websites, ask deeper “how” and “why” questions, and dedicate discretionary time to the topic. This phase reflects a shift from exploration and engagement primarily sustained by external prompts to one increasingly driven by internal motivation. Individuals now generate their own questions and see personal value in finding the answers. However, this emerging interest can still be conditional and somewhat fragile. While the overall affective experience is positive, motivation may temporarily falter when individuals face significant challenges, leading to feelings of frustration or confusion (O’Keefe and Linnenbrink-Garcia, 2014). It is precisely at these moments that targeted scaffolding remains necessary. Unlike the broad support in Phase 2, support here is often about providing specific resources, strategic help to overcome a particular obstacle, or encouragement to persist through difficulty, thereby protecting the still-developing interest (Renninger et al., 2019). The value is forming, but because it has not yet become a stable part of the individual’s identity, the interest requires these favorable conditions to continue to grow.

Phase 4: Well-Developed Personal Interest. This stage represents the full internalization of interest into an enduring disposition. The critical distinction from the emerging phase lies in the robustness and stability of motivation. Individuals with a well-developed interest not only persist through challenges but often thrive on them, viewing them as opportunities for growth. Their deeper and more extensive knowledge base acts as a generative engine, allowing them to perceive nuances, make connections to other domains, and formulate long-term, self-set goals. Their engagement is fully self-sustaining, independent of external support or immediate rewards. Behaviorally, this often manifests as joining communities of practice, mentoring others, or integrating the interest into major life choices, such as career paths or significant hobbies. The personal value associated with the domain is now fully integrated into their sense of identity and self-concept, serving as a source of fulfillment, resilience, and well-being (Ryan and Deci, 2022). They proactively seek out diverse and challenging opportunities not just to learn more, but to more fully express this part of themselves (Shin and Kim, 2019).

### 3.2 From state curiosity to personal interest: satisfaction through iterative cycles

State curiosity is elicited when individuals become aware of a gap between what they know and what they want to know (Loewenstein, 1994). However, the experience of curiosity does not necessarily lead to further engagement or lasting interest. Individuals evaluate both the perceived value of the missing information and their perceived competence in acquiring and processing it. When information is regarded as meaningful and the individual feels capable of resolving the gap, curiosity is more likely to translate into active exploration. The transformation from state curiosity to personal interest relies on iterative cycles of curiosity, information seeking, and resolution, which reinforce expectations of future satisfaction, strengthen perceived competence, and gradually build a more elaborate knowledge network. Over time, these cycles lay the foundation for the emergence of enduring personal interest (Shin and Kim, 2019).

Phase 1: Triggered State Curiosity. State curiosity is triggered when individuals recognize a discrepancy between their existing knowledge and the information they seek (Loewenstein, 1994). Such gaps may arise from violations of expectations, incomplete or ambiguous information, or exposure to novel stimuli. For example, curiosity may be aroused when reading a detective novel without knowing the culprit or when encountering a question with an unknown answer. In this phase, the absence of resolution produces an aversive state, whereas successful resolution is experienced as rewarding.

Phase 2: Evaluation of Information Value and Perceived Competence. Once curiosity is triggered, whether it leads to active information seeking depends on a critical evaluation process. Individuals must weigh the perceived value of the information against their perceived competence to obtain and process it. This evaluative process can be effectively understood through the lens of frameworks like the Expected Value of Control (EVC) theory (e.g., Shenhav et al., 2021). EVC theory suggests that the brain performs an implicit cost-benefit analysis, weighing the expected value of resolving the information gap (e.g., its instrumental utility or anticipated satisfaction) against the perceived cost (e.g., mental effort) of acquiring it. In this context, an individual’s perceived competence directly influences their assessment of the cost–higher competence lowers the anticipated effort. Therefore, information seeking is most likely to proceed when the perceived value of the knowledge is high and a strong sense of competence makes the cost of acquisition seem reasonable. Empirical work supports this, with studies highlighting the role of competence in sustaining curiosity-driven engagement (Lievens et al., 2022; Pekrun and Linnenbrink-Garcia, 2014).

Phase 3: Information Seeking and Iterative Satisfaction. In this phase, individuals actively seek new information and experience satisfaction when their curiosity is resolved. Empirical evidence shows that state curiosity predicts real-world information-seeking behavior, such as during the COVID-19 pandemic, when heightened curiosity was associated with more frequent information searches (Lydon-Staley et al., 2022). A single episode of curiosity and resolution is often insufficient to sustain exploration; instead, repeated episodes accumulate into iterative cycles of curiosity and resolution (Lievens et al., 2022). Satisfaction arises from the resolution of uncertainty through the acquisition of new knowledge (Arnone et al., 2011). Each successful resolution enhances expectations of future satisfaction, strengthens perceived competence, and promotes further engagement. Through these cycles, individuals construct more elaborate knowledge networks, laying the groundwork for the emergence of personal interest (Renninger and Hidi, 2011).

Phase 4: Well-Developed Personal Interest. At this stage, curiosity and interest are integrated and self-sustaining. Individuals continue to explore and spontaneously generate new questions, perpetuating curiosity-resolution iterative cycles. Because personal interest is characterized by substantial subjective value and extensive domain knowledge, individuals employ diverse strategies to deepen their understanding of the content they engage with (Renninger et al., 2019). This process increases both the frequency of curiosity experiences and the satisfaction derived from their resolution (Loewenstein, 1994). Moreover, individuals with well-developed personal interest can identify gaps within their existing knowledge system and activate supplementary information inferred from prior knowledge, even in the absence of external cues (Coenen et al., 2019). In this way, curiosity can be generated through both external and internal means, further enhancing knowledge acquisition and intrinsic satisfaction, thereby promoting voluntary curiosity generation (Murayama, 2022).

### 3.3 The interplay and integration of the two pathways

While the primary goal of this review has been to delineate two distinct developmental pathways for conceptual clarity, a complete account must also consider their potential for dynamic interaction. Although we argue that the core stages within each pathway offer a valid framework for analysis, the relationship between interest and curiosity in practice is not mutually exclusive. As influential reviews on the topic have argued, the distinction between them can be “murky,” as they often co-occur and influence one another (Pekrun, 2019). Therefore, their development is perhaps better conceptualized not as parallel tracks but as a braided river, where the two streams converge and mutually reinforce each other over time.

One primary form of interaction is curiosity serving as a catalyst for situational interest. An episode of state curiosity, triggered by a specific information gap, can lead to a rewarding experience of problem resolution (Rotgans and Schmidt, 2014). If a learning environment provides repeated opportunities for such “curiosity-resolution” cycles, the positive effect associated with these experiences can generalize to the topic itself, thereby triggering and maintaining situational interest.

Conversely, interest provides fertile ground for curiosity to arise. As an individual develops sustained situational interest or an emerging personal interest, they build a substantial knowledge base. This knowledge base enables them to perceive more subtle and complex information gaps that a novice might not notice (Renninger and Hidi, 2011), creating a self-sustaining loop where interest and curiosity mutually deepen one another.

The importance of this cyclical relationship is underscored by emerging theoretical frameworks that aim to model precisely this dynamic interplay (e.g., Tan et al., 2024). This suggests that while each pathway has distinct characteristics, their interaction likely acts as a powerful engine driving the transformation from fleeting motivational states to an enduring disposition.

## 4 Mechanisms in personal interest formation: rewards processing

Rewards seeking is a fundamental driver of human behavior and plays a central role in learning and adaptation. The brain’s rewards circuitry, shaped through evolution, is responsible for processing rewards-related information and regulating behavior to fulfill rewards needs (Hidi, 2016). This circuitry primarily involves the striatum, a component of the basal ganglia, and interconnected regions such as the prefrontal and orbitofrontal cortices, forming the cortico-striatal rewards circuit. Dopamine (DA) serves as a key neurotransmitter within this system (Cohen et al., 2012). In the transition from situational interest and state curiosity to personal interest, rewards processing functions as a core underlying mechanism. Our framework for understanding this mechanism is built upon two major theoretical traditions. The first is Reinforcement Learning (RL) theory, which frames knowledge acquisition itself as an intrinsically rewarding process (e.g., Murayama, 2022). The second is Self-Determination Theory (SDT), which posits that satisfying fundamental psychological needs for autonomy and self-relevance is also inherently rewarding (Ryan and Deci, 2022). This dual-pillar approach guides our subsequent analysis of these two distinct but complementary domains.

### 4.1 Rewards processing in knowledge acquisition

The transformation of situational interest and state curiosity into personal interest can be understood as an iterative reinforcement process, in which repeated experiences of information seeking and knowledge acquisition progressively strengthen and solidify into a stable personal interest. Reinforcement learning theory explains how behavior is shaped by updating discrepancies between expected and actual outcomes (Sutton and Barto, 1998). Murayama (2022) drew on classic reinforcement learning theory to propose that knowledge acquisition itself carries intrinsic rewards value. This idea is consistent with classic motivational accounts, which suggest uncertainty will trigger an “epistemic hunger” for knowledge. Satisfying this hunger by acquiring new information generates a sense of satisfaction and positive effects (Berlyne, 1960). In line with this view, neuroscientific evidence shows that curiosity and interest engage dopaminergic pathways, particularly activation of the ventral striatum, which reinforces exploratory behavior through informational prediction errors–the discrepancy between expected and obtained knowledge (Charpentier et al., 2018).

More specifically, the rewards processing of knowledge acquisition involves the formation of expectations, their updating through prediction errors, and the integration of new information into existing knowledge structures. Before acquiring knowledge, individuals anticipate the value of the information they are about to obtain. This process is governed by the principles of prediction error. Specifically, when the actual informational value exceeds expectations, this generates a positive prediction error (PPE). This PPE triggers a phasic burst of dopamine in the ventral striatum, which serves as a powerful “teach” signal to update the expected value of the behavior and reinforce the exploratory actions that led to it (Murayama, 2022). This increased likelihood of subsequent curiosity and exploration is therefore driven by rewarding, better-than-expected informational outcomes. Conversely, if the obtained information is less valuable than expected, a negative prediction error would occur, leading to a dip in dopamine firing. This would devalue the behavior and make future exploration along that path less likely. Empirical evidence supports this mechanism: Litman et al. (2005) found that curiosity intensifies when individuals feel close to an answer, accompanied by stronger activation in the ventral striatum. Similarly, Lydon-Staley et al. (2022) demonstrated that curiosity-driven exploration relies on the mesolimbic dopamine system, particularly the functional connectivity between the ventral tegmental area and the nucleus accumbens, providing direct neural evidence for the rewards basis of knowledge acquisition. Once obtained, new information can be integrated into prior knowledge, which may reveal further gaps and stimulate renewed curiosity. This recursive cycle, described by Murayama (2022) as a self-boosting effect, illustrates how rewards processing not only reinforces immediate curiosity but also drives the long-term transformation of situational curiosity into enduring personal interest.

### 4.2 Rewards processing in autonomy and self-relevance

While knowledge acquisition itself is rewarding, as discussed in Section “4.1 Rewards processing in knowledge acquisition,” the magnitude and impact of this rewards are not constant. They are powerfully amplified by self-related factors such as autonomy and self-relevance. These factors do not act in isolation; rather, they are crucial catalysts within the developmental pathways, transforming the rewarding experience of learning from a simple cognitive gain into a more profound, personalized, and memorable event that fosters long-term commitment.

Autonomy, the experience of acting with volition and control, enhances the rewards signal by fostering a sense of agency. When individuals feel their actions directly cause an outcome, the rewarding feeling of success is amplified. In the context of our developmental pathways, this mechanism is critical. For example, when an individual in Phase 2 or 3 chooses to explore a topic rather than being assigned it, the subsequent satisfaction from resolving curiosity or understanding a concept is magnified. This amplified rewards more strongly reinforces the exploratory behavior, increasing the likelihood that they will reengage and thus helping to sustain interest through its fragile emerging phase. Neuroscientifically, this is supported by findings that the mere act of making a choice enhances activity in the ventral striatum, even when outcomes are identical, suggesting the brain treats control itself as an intrinsic rewards (Murty et al., 2015).

Furthermore, self-relevance fosters the nature of the rewards by linking new information to an individual’s identity. When information is perceived as personally meaningful, it is not only prioritized cognitively (the “self-reference effect”; Symons and Johnson, 1997) but also endowed with a deeper, personalized value. This is the core process that solidifies personal value, the cornerstone of the transition from maintained situational interest (Phase 2) to a well-developed personal interest (Phase 4). The neural basis for this lies in the profound interaction between the self-referential network (headquartered in the medial prefrontal cortex, mPFC) and the rewards circuit (including the ventral striatum) (Qin and Northoff, 2011). Studies show these regions co-activate when processing information that is both self-relevant and rewarding (Zhan et al., 2016). This neural integration transforms the rewards from “knowing something new” into “knowing something new that matters to me.” It is this personalized and potent rewards signal that provides the robust motivational force needed to build a stable, well-developed personal interest.

## 5 Conclusion

Interest and curiosity are core motivational factors in learning, with stable personal interest important for sustaining engagement and supporting ongoing growth. While personal interest can originate from transient situational interest and state curiosity, not all such states develop into stable forms. This review clarified the conceptual distinctions and commonalities between situational interest and state curiosity and proposed two developmental pathways through which these states may transform into personal interest. The first pathway, from situational interest to personal interest, progresses through four stages: triggered situational interest, maintained situational interest, emerging personal interest, and well-developed, self-sustaining interest. The second pathway, from state curiosity to personal interest, develops through the stages of curiosity triggering, evaluation of information value and perceived competence, information seeking with iterative curiosity satisfaction, and the emergence of a well-developed personal interest. Across both pathways, rewards processing serves as an underlying mechanism, operating through the intrinsic rewards of knowledge acquisition and the motivational benefits of autonomy and self-relevance. Together, these perspectives provide a framework for understanding how fleeting experiences of interest and curiosity can develop into enduring personal interest.

Future research should address four directions. First, the role of trait curiosity in the development of personal interest remains underexplored. Although trait curiosity, as a stable personality characteristic, is correlated with state curiosity (Grossnickle, 2016) and is often interpreted as influencing state curiosity, no studies have examined whether long-term interventions that trigger state curiosity can enhance trait curiosity. Future work could employ extended interventions and computational modeling to clarify the bidirectional relationship between trait and state curiosity, as well as their joint contributions to personal interest development. Second, a lifespan perspective is needed to understand how different types of interest and curiosity emerge, change, and interact over time. Existing evidence suggests that intellectual curiosity shows changes across the lifespan, generally declining from young adulthood into old age, although this trajectory can be influenced by various factors (von Stumm et al., 2011). Regarding interest development, situational interest in childhood is primarily triggered by emotional factors, whereas in adolescence it is often elicited by cognitive factors (Renninger and Hidi, 2016). However, research systematically charting the developmental dynamics of various forms of interest and curiosity across the lifespan is limited. Third, interdisciplinary approaches are needed to advance the study of curiosity and interest. While educational research has focused on developmental processes and practical applications (Renninger and Hidi, 2020) and psychological research has examined their cognitive and behavioral functions, there is a growing need to integrate these with neuroscientific findings (Kidd and Hayden, 2015), neuroscientific studies have only recently begun to investigate the neural basis of curiosity, particularly state curiosity (Cervera et al., 2020). Future research should integrate insights from education, psychology, and neuroscience to develop an integrated model explaining the shared and distinct mechanisms of situational interest and state curiosity, and their developmental pathways to long-term personal interest. Fourth, while the present review has delineated two distinct developmental pathways for conceptual clarity, a critical direction for future research is to investigate their dynamic interplay. In practice, interest and curiosity are unlikely to operate in isolation. An important theoretical question is how these two pathways might influence one another over time. For instance, how repeated cycles of curiosity satisfaction might accumulate to trigger situational interest, and how an established interest, in turn, might provide the knowledge base to generate more sophisticated forms of curiosity. Exploring this bidirectional relationship will be a key step toward a more comprehensive, integrated model of personal interest formation and will likely require longitudinal and micro-analytic research designs.
