# Correction: From situational interest and state curiosity to personal interest: developmental pathways and underlying mechanisms

**DOI:** 10.3389/fpsyg.2026.1789574

**Published:** 2026-01-29

**Authors:** Yuling Yan, Yilei Jia, Yuxin Li, Feiyi Liu, Mingxia Zhang

**Affiliations:** 1 Institute of Psychology, Chinese Academy of Sciences, Beijing, China; 2 Department of Psychology, University of Chinese Academy of Sciences, Beijing, China

**Keywords:** interest, curiosity, knowledge acquisition, self-relevance, rewards processing

The order of the affiliations of the published paper was erroneously given as:

1. Department of Psychology, University of Chinese Academy of Sciences, Beijing, China
2. Institute of Psychology, Chinese Academy of Sciences, Beijing, China

The correct affiliations list reads:

1. Institute of Psychology, Chinese Academy of Sciences, Beijing, China
2. Department of Psychology, University of Chinese Academy of Sciences, Beijing, China

Affiliation Institute of Psychology, Chinese Academy of Sciences, Beijing, China was omitted for author Mingxia Zhang. This affiliation has now been added for author Mingxia Zhang.

Author Mingxia Zhang was erroneously assigned to affiliation Department of Psychology, University of Chinese Academy of Sciences, Beijing, China. This affiliation has now been removed for author Mingxia Zhang.

The original version of this article has been updated.

